# Effect of acupressure on pain intensity and physiological indices in patients undergoing extracorporeal shock wave lithotripsy: a randomized double-blind sham-controlled clinical trial

**DOI:** 10.1186/s12906-024-04360-1

**Published:** 2024-01-25

**Authors:** Ali Safdari, Salman Khazaei, Mahdi Biglarkhani, Seyed Habibollah Mousavibahar, Seyed Reza Borzou

**Affiliations:** 1 Student Research Committee, Hamadan University of Medical Sciences, Hamadan, Iran; 2 Department of Epidemiology, Research Center for Health Sciences, Hamadan University of Medical Sciences, Hamadan, Iran; 3 Department of Persian Medicine, School of Medicine, Hamadan University of Medical Sciences, Hamadan, Iran; 4 Urology & Nephrology Research Center, Hamadan University of Medical Sciences, Hamadan, Iran; 5 Department of Medical Surgical Nursing, School of Nursing and Midwifery, Chronic Diseases (Home Care) Research Center, Hamadan University of Medical Sciences, Hamadan, Iran

**Keywords:** Acupressure, Pain, Physiological indices, Lithotripsy, Renal stones, Complementary therapy

## Abstract

**Background:**

Despite the widespread use of extracorporeal shock wave lithotripsy (ESWL) as a treatment for kidney stones, it is essential to apply methods to control pain and improve patient comfort during this procedure. Therefore, this study aimed to investigate the effect of acupressure at the Qiu point on pain intensity and physiological indices in patients undergoing ESWL.

**Methods:**

This randomized, sham-controlled clinical trial was conducted at the Shahid Beheshti Educational-medical Center in Hamadan City (western Iran) from May to August 2023. Seventy-four eligible patients were split into intervention (*n* = 37) and sham (*n* = 37) groups. Ten minutes before lithotripsy, the intervention group received acupressure at the Qiu point, while the sham group received touch at a neutral point. The primary outcomes were pain intensity measured by the Visual Analog Scale (VAS) and physiological indices such as blood pressure and heart rate at baseline, 1, 10, 20, 30, 40, and 50 min after the intervention. The secondary outcomes included lithotripsy success and satisfaction with acupressure application.

**Results:**

The analysis of 70 patients showed no significant differences in the demographic and clinical information of the patients across the two groups before the study (*P* > 0.05). Generalized estimating equations revealed that the interaction effects of time and group in pain and heart rate were significant at 30 and 40 min (*P* < 0.05). The results of this analysis for systolic blood pressure revealed a significant interaction at 30 min (*P* = 0.035). However, no significant interaction effects were found for diastolic blood pressure changes (*P* > 0.05).

**Conclusions:**

Acupressure at the Qiu point positively impacts pain in patients undergoing ESWL treatment and increases their satisfaction. However, these results for physiological indices require further studies. Thus, acupressure can be considered a simple, easy, and effective option for pain management in patients during this procedure.

**Trial registration:**

[https://en.irct.ir/trial/69117], identifier [IRCT20190524043687N4].

## Background

Urinary stones are one of the most common reasons individuals seek urology clinics [1], affecting 12% of the general population [2]. Extracorporeal shock wave lithotripsy (ESWL), a non-invasive therapeutic technique using shock waves, is considered the standard treatment for upper urinary tract and kidney stones [3]. Since its first reported use in 1984 [4], ESWL has quickly gained popularity as an alternative to invasive surgeries due to its non-invasiveness [3]. The ESWL is the first-line treatment for kidney stones smaller than 2 cm [5]. However, this method inherently causes significant pain; thus, pain relief is recommended for patients during the procedure [6]. Severe pain might limit the opportunity for applying sufficient energy doses, leading to additional movement and increased respiration rates [7]. Additionally, acute pain may increase the risk of kidney hematoma following stone fragmentation due to increased blood pressure [1].

Pain sources from ESWL include somatic pain (superficial pain resulting from the impact of waves on the skin and muscles) [2] and visceral pain (deep pain resulting from the penetration of shock waves into the capsule and nerves of the kidney) [5]. There is no consensus on a standardized analgesic regimen for optimal pain reduction during lithotripsy [8]. On the one hand, the use of pharmacological regimens, such as nonsteroidal anti-inflammatory drugs (NSAIDs) and opioid medications, during lithotripsy is associated with limitations [9]. Undesirable effects of NSAIDs include allergy, gastrointestinal bleeding, decreased renal blood flow, and even kidney failure [10]. Additionally, the use of opioids may cause various side effects such as nausea, vomiting, low blood pressure, and respiratory depression [11]. Therefore, it is essential to develop non-pharmacological methods to control vital signs and reduce patient pain [12].

In recent years, non-pharmacological methods such as acupressure and acupuncture have gained significant attention [13]. The application of hand and finger pressure at acupuncture points in acupressure is believed to lead to a balanced energy system and a stable state of homeostasis in the body [14]. According to Western theory, the analgesic effect of acupressure can be explained through the endorphin theory (release of a chemical substance effective in pain) and the gate control theory (blocking the transmission of pain signals to higher nerve centers following acupressure stimulation) [15]. Furthermore, acupressure results in pain relief by vasodilation and increasing blood flow, secretion, and release of chemicals such as dopamine and serotonin [16]. Experimental and clinical studies have well supported the effects of acupressure on pain relief, stabilization of physiological indicators, and reduction in analgesic consumption [13, 17]. Also, it has been reported that there is a direct correlation between pain and patient satisfaction [18], and patients undergoing ESWL may need repeated treatment sessions [19]. This non-pharmacological method is effective, safe, and cost-effective, providing pain relief with patient satisfaction without additional equipment [12, 20].

To the best of our knowledge, there have been limited studies on the impact of acupressure on managing complications during ESWL. On the other hand, some available evidence has reported contradictory results regarding the effectiveness of acupressure in different clinical situations [11, 13]. In this study, acupressure at the Qiu point is proposed, and its effectiveness in alleviating colicky pains has been documented in several studies [21, 22]. No studies have been conducted in the existing literature on using acupressure at the Qiu point to manage pain and physiological indices in patients undergoing ESWL, highlighting the necessity for research in this area. Additionally, there appears to be no evaluation of the effects of acupressure on lithotripsy success and patient satisfaction. Considering the importance of managing complications during lithotripsy and the growing interest in complementary therapies, this study aims to examine the impact of acupressure at the Qiu point on pain intensity and physiological indices in ESWL patients. Moreover, patients' satisfaction with acupressure application and the success of lithotripsy were evaluated as secondary outcomes.

## Methods

### Study design and setting

**Fig. 1:**
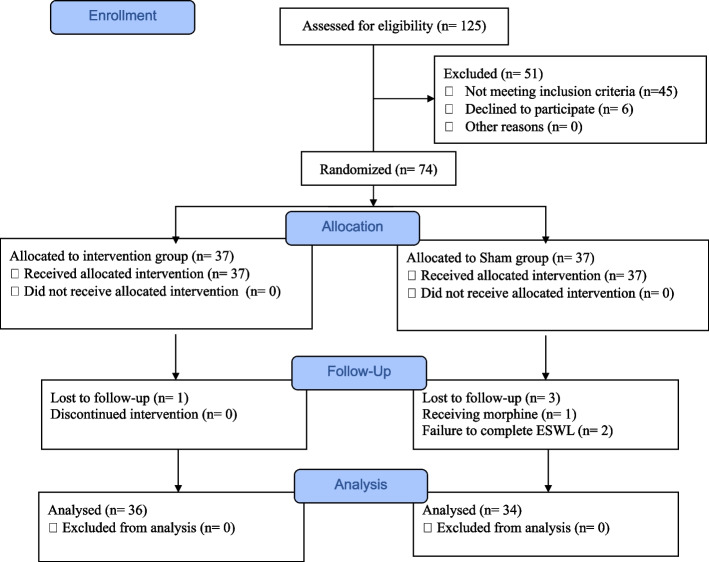
CONSORT flowchart of the research

This randomized, single-center, and double-blind sham-controlled clinical trial was conducted at the Shahid Beheshti Educational-medical Center in Hamadan City, western Iran, from May to August 2023. The study was designed and carried out according to the Standards for Reporting Interventions in Controlled Trials of Acupuncture (STRICTA) guidelines [23] and the CONSORT statement [24]. Figure 1 depicts the flowchart of this clinical trial.

### Study participants

The researchers selected participants for the study based on the European Urology Association's guidelines for ESWL [6]. Inclusion criteria were the signing of an informed consent form, age between 18 and 65 years, diagnosis of kidney stones (stones smaller than 2 cm), and undergoing ESWL for the first time. Exclusion criteria included patients with multiple and radiolucent stones, double J stents, kidney abnormalities, severe hydronephrosis, acute urinary tract infection, emergency lithotripsy, BMI over 30 kg/m^2^, substance abuse, anticoagulant drug use, having an obstruction at the acupressure site, and receiving analgesics 12 h before lithotripsy. According to the center's strategy, analgesics were not routinely administered; however, in case of unbearable pain, intravenous morphine (0.1 mg/kg) was administered. Participants were informed that they could ask for analgesics if the pain was unbearable, and those who made such requests were excluded from the study.

### Pilot study

In the pilot assessment phase of the study, methodological procedures and the assurance of the minimal non-specific effects of the sham point were evaluated before the main samples. The validity of the correct locations of acupressure points and the quality of the massage were confirmed through concurrent validity determination. After conducting the pilot study, the researchers concluded that the intervention should be performed twice before lithotripsy, each time for 3 min. The time intervals for pain outcome measurements were altered from 5-time breaks to 7-time intervals (before administration, exactly before performing ESWL, and 10, 20, 30, 40, and 50 min after the start of treatment). Based on the pilot study results, the chosen sham point did not impact the study outcomes.

### Sample size calculation

Based on a previous study, the sample size was determined using G-Power software [16]. Considering a type I error of 0.05, a test power of 90%, and an effect size of 0.8, the calculated sample size was 33 participants for each group. With an anticipated 10% dropout rate, 37 participants were included in each group for the study.

### Randomization and blinding

A block randomization method with a block size of four was employed in this study. The permutations were generated and selected using a computer. Randomization and blinding were performed by a professional statistician outside the research team to ensure the unpredictability of allocating individuals to groups. This sequence remained concealed until the completion of interventions. The concealment of randomization was achieved using opaque and wax-sealed envelopes numbered with a random sequence. Shortly before the intervention, the envelopes were sequentially opened for eligible patients, revealing the intervention group assignment. In this study, the blinded groups included the patients, research coordinator, radiography unit of the lithotripsy department, urology specialist, and outcome analyzer. The results of the study were evaluated by a research colleague who was unaware of the allocation of patients to the intervention and sham groups. Additionally, the radiography unit was aware of an ongoing acupressure study but remained blinded to the hypothesis and protocol of the study. To prevent interactions between participants in different groups, patients were placed in separate rooms during the intervention and entered the lithotripsy ward individually.

### Interventions

The acupressure protocol used in the study was designed and implemented based on previous studies [15, 25], the pilot study findings, and the clinical experience of the acupuncture specialist in the team. The acupressure intervention in both the intervention and sham groups was applied at the Qiu point by a trained individual (the first author). This point was first identified by Yunqiao Qiu, a urology specialist at a hospital affiliated with Guangzhou Medical University in China, for treating acute kidney colic pain [22]. This anatomical point is located about the width of one finger (the size of the body is equivalent to 1.3 inches) below and one thumb's width inside the intersection of the twelfth rib and the column of vertebrae (Lumbo-costal point) [21].

Patients in the intervention group received the intervention 10 min before ESWL in the same location in a room with minimal environmental stimuli (including sound, temperature, and light) while maintaining privacy. To accomplish this intervention, the researcher after washing and warming their hands, asked the patients to lie on the side opposite the painful area, bending their knees towards the abdomen. The acupressure intervention was gradually applied using fingertip pressure in a calm, rhythmic manner until participants reported sensations of warmth, heaviness, numbness, and pain in the target area [15]. Various studies have suggested a massage duration of 2–5 min to achieve desired outcomes [25], and our preliminary study results supported this time range. Hence, a 3-min massage intervention was administered in the current study. Additionally, the massage intervention was repeated once exactly before the commencement of lithotripsy. Patients in the sham group received superficial touches at a neutral point and its surrounding areas according to the protocol. Based on the findings of previous studies, non-acupoints should be approximately 2.5–4 cm away from the current acupuncture point [26, 27]. Patients in this group reported no sensations of pain or pressure [15, 28]. The pilot study confirmed the lack of therapeutic effect of this point on primary outcomes in patients undergoing ESWL treatment. Table 1 presents the details of the enrollment, interventions, and evaluations.

**Table 1:**
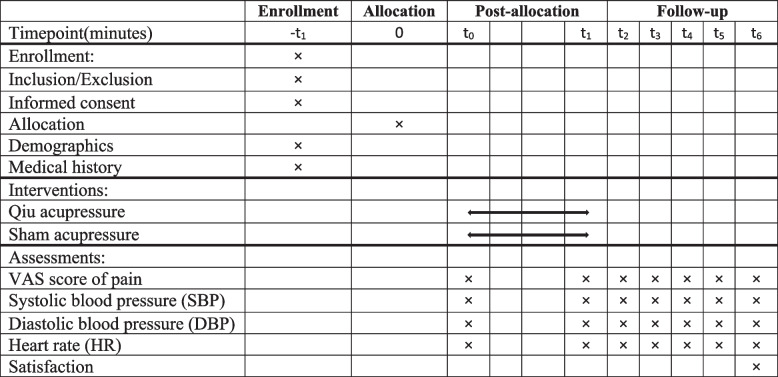
The schedule of enrollment, interventions and assessments

### Lithotripsy protocol

All patients in this study underwent treatment using the HK. ESWL-V extracorporeal lithotripsy system (Wikkon, Shenzhen, China) under the supervision of an experienced radiograph and the urology specialist (SHM). Patients were positioned prone and underwent treatment while guided by fluoroscopy (to locate the stone). The ultrasonography gel was used as a coupling agent for ESWL. The treatment process started with low energy at a voltage of 10 kW (in the first 500 shocks) and gradually increased based on the patient's tolerance. The treatment goal was to deliver 3200–4000 shock waves at an energy level of 12–16 kW, aiming for maximum kidney stone clearance, per the operator's experience with several years of lithotripsy device usage. Treatment sessions lasted approximately 30- 40 min. The initial stone diagnosis was based on kidney ultrasonography and kidney-ureter-bladder (KUB) radiography.

### Outcomes

#### Clinical and demographic information forms

Demographic information included age, gender, education, body mass index (BMI), and marital status. Clinical information comprised stone parameters (side, location, and size) and the intensity of each treatment based on the maximum energy level (kV), the number of shocks applied (Shock numbers), and the duration of the treatment recorded.

#### Visual analog scale (VAS)

Patient pain was measured using the Visual Analog Scale (VAS), developed by Price et al. in 1983 [29]. This scale consists of a graded line ranging from 0 to 10. Due to its ease of use and quick evaluation of results, VAS has become the most suitable tool for measuring acute pain severity [16]. and its reliability has been confirmed in various studies [12, 30]. Pain intensity in patients was assessed before massage and at 1, 10, 20, 30, 40, and 50 min after lithotripsy.

#### Measurement of physiological indexes

Blood pressure in patients was measured using the Zenit Mod LD-579 arm blood pressure monitor. The evaluation of physiological indices, including systolic and diastolic blood pressures and heart rate, is detailed in Table 1.

#### Success of ESWL

Three weeks after lithotripsy, the success of stone clearance was examined through follow-up evaluation with ultrasonography. Stones smaller than 4 mm were classified as stone-free [5].

#### Satisfaction of interventions

Patients' satisfaction with the acupressure intervention was assessed as a variable on a five-point Likert scale from 1 = (very dissatisfied) to 5 = (very satisfied).

### Statistical analysis

Data analysis for this study was conducted using Stata software (version 14). The report included descriptive statistics, such as mean and standard deviation, presented in graphs and statistical tables. The Chi-square test was applied to categorical data to examine group homogeneity, and the independent t-test was applied to continuous data. The study utilized Generalized Estimating Equations (GEE) and analytical statistics to explore differences between the intervention and sham groups in terms of pain outcomes and physiological indices at a significance level of 0.05.

## Results

### Recruitment

Out of 125 eligible patients for the study, 45 patients did not meet the inclusion criteria, and six patients and hence were excluded from randomization. Consequently, 74 eligible patients were equally (*n* = 37) allocated to the intervention and sham groups. During the follow-up period, four patients dropped out after randomization. Thus, the final analysis was conducted on 70 patients. The results did not demonstrate significant differences between the two groups in terms of age, gender, BMI, stone characteristics (size, location, and side), and treatment parameters (the number of shock waves, energy, and duration) (*p* > 0.05) (Table 2).

**Table 2:** Baseline demographic and clinical characteristics of participants

Characteristics	Intervention group (*n* = 36)	Sham group (*n* = 34)	*P* value^d^
**Gender, no. (%)**			0.238^a^
Male	26 (72.22%)	20 (58.82%)	
Female	10 (27.78%)	14 (41.18%)	
**Level of Education, no. (%)**			0.731^c^
Primary school	11 (30.56%)	12 (35.29%)	
Middle school	5 (13.89%)	5 (14.71%)	
Diploma	14 (38.89%)	11 (32.35%)	
Undergraduate	4 (11.11%)	6 (17.65%)	
Graduate	2 (5.56%)	0 (0%)	
**Marital status, no. (%)**			0.868^a^
Single	8 (22.22%)	7 (20.59%)	
Marid	28 (77.78%)	27 (79.41%)	
**Stone site, no. (%)**			0.484^a^
Right	15 (41.67%)	17 (50%)	
Left	21 (58.33%)	17 (50%)	
**Stone location, no. (%)**			0.823^c^
Upper Calix	7 (19.44%)	5 (14.71%)	
Middle Calix	3 (8.34%)	6 (17.64%)	
Lower Calix	10 (27.78%)	10 (29.41%)	
Pelvis	9 (25%)	8 (23.53%)	
Upper ureter	7 (19.44%)	5 (14.71%)	
**Location, no. (%)**			0.094^c^
City	26 (72.22%)	30 (88.24%)	
Village	10 (27.78%)	4 (11.76%)	
**Success of ESWL, no. (%)**			0.673^a^
Yes	11 (30.56%)	12 (35.29%)	
No	25 (69.44%)	22 (64.71%)	
**Satisfaction, no. (%)**			0.556^c^
Very satisfied	10 (27.78%)	8 (23.53%)	
Satisfied	12 (33.33%)	8 (23.53%)	
Neither	10 (27.78%)	13 (38.24%)	
Dissatisfied	4 (11.11%)	3 (8.82%)	
Very dissatisfied	0 (0%)	2 (5.88%)	
**BMI (kg/m2), mean (SD)**	25.10 (2.98)	25.06 (2.68)	0.961^b^
**Age (year), mean (SD)**	44.75 (11.13)	44.23 (12.94)	0.858^b^
**Number of shocks, mean (SD)**	3552.22 (215.95)	3531.85 (229.75)	0.703^b^
**Voltage (kV), mean (SD)**	14.72 (0.84)	14.73 (0.75)	0.945^b^
**Stone size(mm), mean (SD)**	11.22 (3.24)	11.08 (3.16)	0.861^b^
**ESWL duration (min), mean (SD)**	34.08 (2.81)	33.64 (3.19)	0.546^b^

*Abbreviations*: *no* number, *SD* standard deviation, *BMI* body mass index, *min* minutes, *mm* millimeters

^a^ Chi square test

^b^ independent-samples t-test

^c^ Fisher's exact test

^d^ *p* < 0.05 was considered statistically significant

Approximately 66% of the samples were male. The mean ages of participants in the intervention and control groups were 44.75 ± 11.13 and 44.23 ± 12.94 years, respectively. The stone size averaged 11.22 ± 3.24 and 11.08 ± 3.16 mm in the intervention and control groups, respectively. In this study, the lower calyx and pelvis were the most common locations for stone formation among the patients. The numbers of shock waves in the intervention and sham groups were 3552.22 ± 215.95 and 3531.85 ± 229.75, respectively (*P* = 0.703). The mean duration of lithotripsy in the intervention and sham groups was 34.08 ± 2.81 and 33.64 ± 3.19, respectively (*P* = 0.546), and the mean energy value was 14.72 ± 0.84 and 14.73 ± 0.75, respectively (*P* = 0.945) (Table 2). No adverse effects associated with acupressure were reported in the intervention and sham groups.

### Clinical outcomes

Tables 3 and 4 present the results of the GEE analysis comparing the intervention and sham groups in terms of pain, heart rate, and systolic/diastolic blood pressure at different time points. Pain intensity significantly changed over time, especially at 20, 30, and 40 min after the intervention (*P* < 0.05). The interaction of group and time showed significant differences in pain only at 20 and 30 min (*P* < 0.05). The study also revealed significant heart rate changes, 30 and 40 min after the intervention (*P* < 0.05). The interaction of group and time revealed significant differences in heart rate at 20 and 30 min (*P* < 0.05) (Table 3).

**Table 3:** Generalized estimating equation (GEE) for pain intensity and heart rate (*N* = 70)

Variables	Pain		Heart rate	
	Estimate (95% CI)	*P*-value*	Estimate (95% CI)	*P*-value
Group	0.088 (-0.572 to 0.748)	0.793	1.761 (-3.143 to 6.666)	0.482
Follow‐up time				
t_1_ (1 min vs. pre‐test)	-0.176 (-0.763 to 0.410)	0.556	-2.588 (-6.503 to 1.327)	0.195
t_2_ (10 min vs. pre‐test)	0.176 (-0.410 to 0.763)	0.556	-3.676 (-7.591 to 0.238)	0.066
t_3_ (20 min vs. pre‐test)	2.058 (1.471 to 2.645)	**< 0.001**	2.323 (-1.591 to 6.238)	0.245
t_4_ (30 min vs. pre‐test)	4.382 (3.795 to 4.969)	**< 0.001**	16.147 (12.231 to 20.062)	**< 0.001**
t_5_ (40 min vs. pre‐test)	0.617 (0.030 to 1.204)	**0.039**	6.205 (2.290 to 10.121)	**0.002**
t_6_ (50 min vs. pre‐test)	-0.058 (-0.645 to 0.528)	0.844	-0.470 (-4.386 to 3.444)	0.814
Group × Time				
Group × 1 min	-0.267 (-1.086 to 0.550)	0.521	-2.245 (-7.704 to 3.214)	0.420
Group × 10 min	-0.398 (-1.217 to 0.419)	0.340	-2.656 (-8.116 to 2.802)	0.340
Group × 20 min	-0.919 (-1.738 to -0.101)	**0.028**	-6.740 (-12.20 to -1.280)	**0.016**
Group × 30 min	-1.437 (-2.256 to -0.619)	**0.001**	-8.397 (-13.856 to -2.937)	**0.003**
Group × 40 min	-0.450 (-1.269 to 0.367)	0.280	-2.761 (-8.221 to 2.698)	0.322
Group × 50 min	-0.607 (-1.426 to 0.210)	0.146	-3.640 (-9.10 to 1.819)	0.191

^*^p < 0.05 was considered statistically significant; Boldface indicates the *P* value is significant

**Table 4:** Generalized estimating equation (GEE) for systolic blood pressure and diastolic blood pressure (*N* = 70)

Variables	Systolic blood pressure		Diastolic blood pressure	
	Estimate (95% CI)	*P*-value^*^	Estimate (95% CI)	*P*-value
Group	-0.841 (-7.604 to 5.921)	0.807	-0.575 (-5.347 to 4.197)	0.813
Follow‐up time				
t_1_ (1 min vs. pre‐test)	-6.205 (-11.097 to -1.314)	**0.013**	-1.852 (-4.394 to 0.688)	0.153
t_2_ (10 min vs. pre‐test)	-6.088 (-10.979 to -1.196)	**0.015**	-3.117 (-5.659 to -0.576)	**0.016**
t_3_ (20 min vs. pre‐test)	-3.029 (-7.921 to 1.862)	0.225	-2.529 (-5.070 to 0.012)	0.051
t_4_ (30 min vs. pre‐test)	12.058 (7.167 to 16.950)	**< 0.001**	5.235 (-2.693 to 7.776)	**< 0.001**
t_5_ (40 min vs. pre‐test)	-0.647 (-5.538 to 4.244)	0.795	-0.323 (-2.864 to 2.217)	0.803
t_6_ (50 min vs. pre‐test)	-8.0 (-12.891 to -3.108)	**0.001**	-4.882 (-7.423 to -2.340)	**< 0.001**
Group × Time				
Group × 1 min	-2.488 (-9.309 to 4.332)	0.475	-1.591 (-5.135 to 1.952)	0.379
Group × 10 min	-2.745 (-9.566 to 4.076)	0.430	-0.271 (-3.815 to 3.272)	0.881
Group × 20 min	0.501 (-6.319 to 7.322)	0.885	1.473 (-2.070 to 5.017)	0.415
Group × 30 min	-7.336 (-14.157 to -0.515)	**0.035**	-3.235 (-6.779 to 0.308)	0.074
Group × 40 min	-0.825 (-7.646 to 5.995)	0.813	0.490 (-3.053 to 4.034)	0.786
Group × 50 min	-0.583 (-7.404 to 6.237)	0.867	-0.367 (-3.911 to 3.176)	0.839

^*^p < 0.05 was considered statistically significant; Boldface indicates the *P* value is significant

**Fig. 2:**
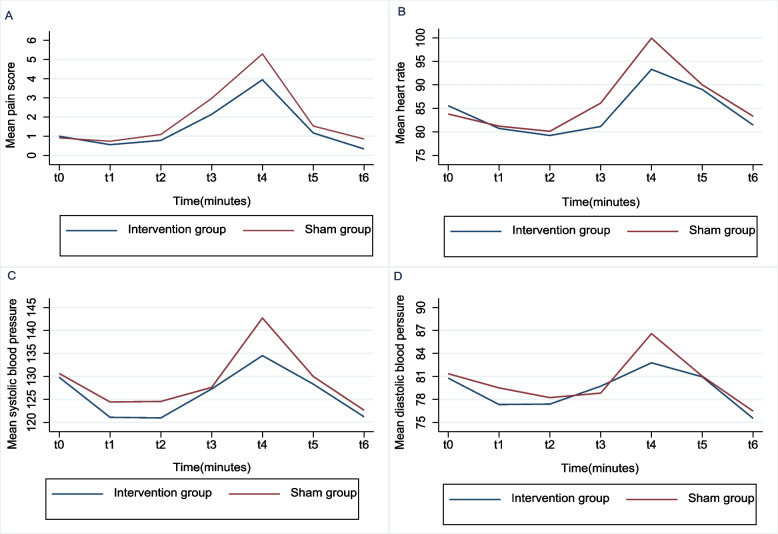
Two-way line charts comparing acupressure versus sham (**A**) in intensity of pain and each physiological index: (**B**) Heart rate, (**C**) Systolic blood pressure, (**D**) Diastolic blood pressure

Moreover, significant differences were observed between the pre‐test and post-test results at 1, 10, 30, and 50 min for systolic blood pressure and 10, 30, and 50 min for diastolic blood pressure (*p* < 0.05) (Fig. 2). However, the interaction effects between the group and time were not significant on diastolic blood pressure changes (*P* > 0.05). In contrast, this interaction was significant for systolic blood pressure at 30 min (*P* = 0.035) (Table 4).

### Other findings

Over 54% of patients reported that they were very satisfied or satisfied with the acupressure intervention., whereas only 2.85% of the participants were very dissatisfied with the implementation method. Furthermore, the data analysis showed no significant relationships between gender, assigned group, and the success of stone fragmentation (*P* > 0.05).

## Discussion

This clinical trial examined the effect of acupressure on pain intensity and physiological indicators in patients treated with ESWL. It was found that massaging the Qiu point for 10 min before ESWL reduced pain and increased patient satisfaction.

In our study, the pain-relieving effects of acupressure were not significant at the initial stages of lithotripsy, probably due to the mild pain at the start of treatment with low energy. The more significant reduction in pain at later stages in the acupressure group might be attributed to more time needed to release chemicals and adjust energy flow [16]. According to a meta-analysis in 2023, the potent analgesic effect of acupuncture requires 15–30 min [10]. However, one study found that massaging the Qiu point provided quicker pain relief than sodium parecoxib in the first 10 min [21]. This difference may be related to pain pathogenesis during lithotripsy compared to colicky pain. Stimulating surface pain receptors in the skin and visceral receptors in the peritoneum, muscles, periosteum, and renal capsule are potential sources of pain during ESWL [31].

Traditional Chinese Medicine suggests acupressure clears blockages in meridian channels, restoring balance to the nervous system [32]. The Western theory explains the effect of acupressure on pain reduction through the endorphin theory (the release of pain-relieving chemicals) and the gate control theory (blocking pain message transmission to higher nerve centers following acupressure stimulation) [15]. Moreover, considering psychological pain mechanisms, manually massaging acupuncture points not only adjusts patients' mental states but also reduces pain-causing mediators [33]. Various clinical evidence supports the impact of acupressure in reducing patient pain [16, 17, 25]. Şolt Kirca et al. showed that the pain-relieving effects of acupressure at points LV4 and LI4 last for about 120 min [16]. Corresponding to these findings, Düzel et al. (2023) reported beneficial effects in reducing pain in patients after angiography [34]. However, one study did not find a significant effect of acupressure on the pain of colic patients [11]. The lack of previous studies on the impact of acupressure on pain during ESWL limits the opportunity for further comparison and discussion.

Insufficient pain relief during ESWL leads to undesirable patient movements and increased respiratory effort. This hampers the concentration of energy on the stone and reduces the success of lithotripsy [3]. The lithotripsy operator may also reduce the device's frequency and voltage due to the patient's unbearable and severe pain, resulting in a proportional decrease in lithotripsy success [35]. The most commonly used drug regimens for this purpose include NSAIDs such as diclofenac [36], sodium parecoxib [21], and opioids such as morphine [2]. However, the use of these drugs may be associated with several adverse effects: reduced renal blood flow, renal failure, severe nausea, vomiting, and potentially dangerous complications like respiratory depression, in addition to their high costs [10, 18]. In contrast to the results obtained from drug regimens, studies have reported pain relief resulting from acupressure without adverse effects or with minor side effects [12, 25, 30]. This study examined the impact of acupressure at the Qiu point. This acupuncture point has been identified and introduced recently for relieving colicky pain [22]. The existing evidence reports promising effects on pain relief in patients. Chen et al. showed that the pain-relieving effects of Qiu point massage were faster and more effective than sodium paracoxib in the first 10 min of intervention [21]. The results of a meta-analysis also highlighted the effectiveness of this massage compared to standard treatments for colicky pain [37]. Also, several previous studies have randomly found that cures in the areas around this point are effective in colicky pain. For example, Gul and Gul reported similar pain relief effects after sterile water injection into the restricted triangular area bordered by the 12th rib, the spinal column, and the iliac crest, akin to an intramuscular diclofenac injection [9]. Maldonado Avila et al. found that the infusion of 2% lidocaine into the twelfth thoracic nerve could effectively relieve kidney stone pain [36].

Nociceptive effects are associated with increased blood pressure, respiratory rate, and heart rate [34]. Pain during ESWL increases the risk of kidney hematoma with increased blood pressure [7]. Our study revealed significant effects of acupressure on patients' systolic blood pressure during the peak of shockwave energy, which is not consistent with the study of Düzel et al. [34]. Khoram et al. also found that massaging three points, HT7, EX-HN3, and GB20, effectively reduced anxiety, heart rate, and systolic blood pressure in patients before open-heart surgery [13]. The difference may be due to the different locations of the acupressure points and the employment of multiple points [34], in contrast to a single point used for massage in our study. Differences in outcome assessment duration, sample size, and cultural characteristics of research samples [13] are the other potential reasons for this difference. Moreover, the results of some previous studies are consistent with our findings. Also, Sharifi Rizi et al. found that massaging the HT7 and LI4 points for two minutes effectively reduced pain intensity in patients undergoing bone marrow biopsy. However, it did not significantly affect blood pressure levels. Researchers attributed this result to the short duration of massage and the sympathetic effects caused by anxiety [12]. A study on pain and vital signs resulting from venipuncture did not report the practical impact of acupressure on patients' vital signs [30]. However, the gate control theory justifies the immediate effects of acupressure [20].

The study findings indicated a statistically significant difference in patients' heart rates between 20 and 30-min time intervals after ESWL started. However, these changes were not clinically significant. There was no statistically significant difference in diastolic blood pressure values reported at any time interval. Similarly, Resim et al. did not find a statistically significant difference in the effects of combined midazolam and tramadol with electroacupuncture on patients' heart rates and blood pressure [38]. It is challenging to achieve physiological changes in a short period during painful procedures because the altered values quickly return to baseline levels after the procedure [30]. Therefore, physiological indicators may not be influenced by acupressure interventions. However, further evidence-based studies are required to obtain more precise results.

In the present study, the stone-free rate was 32.86 percent. Success rates in stone fragmentation have been reported at various levels in different studies [39]. Differences in treatment duration, type of device, and study design are all potential sources affecting the variation in the study [6, 40]. Acupressure intervention also increased patient satisfaction. Consistent with our findings, a study by Yildirim et al. (2021) showed that acupressure reduced venipuncture pain and increased patient satisfaction [30]. The results of a meta-analysis also yielded similar findings [18]. According to the findings of the current research, acupressure at the Qiu point does not significantly influence the physiological indices of patients during ESWL. Nevertheless, applying this method as a safe and cost-effective approach leads to pain reduction and increased patient satisfaction during this procedure.

## Conclusion

The findings of this study demonstrate that acupressure facilitates better tolerance of ESWL by reducing patients' pain levels. Simultaneously, increases their satisfaction with this intervention method. Therefore, it provides the possibility of delivering higher-energy shock waves for achieving stone-free outcomes. This method is non-invasive, simple, affordable, and easy for pain management in patients. However, definitive conclusions about the impact of acupressure on managing ESWL complications require further multi-center studies with larger sample sizes.

### Limitations

This study has several limitations. Researchers used a subjective pain scale to assess patients, which individual differences, cultural factors, and mental and psychological aspects can influence. This variability makes it challenging for researchers to control. Future studies should consider alternative, more objective methods to measure patient pain. The study design did not allow for evaluating the composition of the patients' stones. Other limitations of the study include its small sample size and its implementation at a single center. Due to the variation among different generations of lithotripsy devices and their potential impact on patient pain levels, further research is necessary to compare pain associated with different lithotripsy devices.

## Data Availability

The datasets used and/or analyzed during the current study are available from the corresponding author upon reasonable request.

## Abbreviations

ESWL: Extracorporeal Shock Wave Lithotripsy
GEE: Generalized Estimating Equations
VAS: Visual Analogue Scale
BMI: Body Mass Index
